# In Situ Ultra-Small- and Small-Angle X-ray Scattering Study of ZnO Nanoparticle Formation and Growth through Chemical Bath Deposition in the Presence of Polyvinylpyrrolidone

**DOI:** 10.3390/nano13152180

**Published:** 2023-07-26

**Authors:** Karina Abitaev, Petia Atanasova, Joachim Bill, Natalie Preisig, Ivan Kuzmenko, Jan Ilavsky, Yun Liu, Thomas Sottmann

**Affiliations:** 1 Institute of Physical Chemistry, University of Stuttgart, 70569 Stuttgart, Germany; karina.abitaev@ipc.uni-stuttgart.de (K.A.); natalie.preisig@ipc.uni-stuttgart.de (N.P.); 2 Institute for Materials Science, University of Stuttgart, 70569 Stuttgart, Germany; atanasova@imw.uni-stuttgart.de (P.A.); bill@imw.uni-stuttgart.de (J.B.); 3 X-ray Science Division, Advanced Photon Source, Argonne National Laboratory, Argonne, IL 60439, USA; kuzmenko@anl.gov (I.K.); ilavsky@anl.gov (J.I.); 4 National Institute of Standards and Technology Center for Neutron Research, Gaithersburg, MD 20899, USA; yun.liu@nist.gov

**Keywords:** chemical bath deposition, ZnO, USAXS, SAXS, polyvinylpyrrolidone, PVP, particle formation, kinetics

## Abstract

ZnO inverse opals combine the outstanding properties of the semiconductor ZnO with the high surface area of the open-porous framework, making them valuable photonic and catalysis support materials. One route to produce inverse opals is to mineralize the voids of close-packed polymer nanoparticle templates by chemical bath deposition (CBD) using a ZnO precursor solution, followed by template removal. To ensure synthesis control, the formation and growth of ZnO nanoparticles in a precursor solution containing the organic additive polyvinylpyrrolidone (PVP) was investigated by in situ ultra-small- and small-angle X-ray scattering (USAXS/SAXS). Before that, we studied the precursor solution by in-house SAXS at *T* = 25 °C, revealing the presence of a PVP network with semiflexible chain behavior. Heating the precursor solution to 58 °C or 63 °C initiates the formation of small ZnO nanoparticles that cluster together, as shown by complementary transmission electron microscopy images (TEM) taken after synthesis. The underlying kinetics of this process could be deciphered by quantitatively analyzing the USAXS/SAXS data considering the scattering contributions of particles, clusters, and the PVP network. A nearly quantitative description of both the nucleation and growth period could be achieved using the two-step Finke–Watzky model with slow, continuous nucleation followed by autocatalytic growth.

## 1. Introduction

The field of artificial, structured organic–inorganic hybrid materials has received enormous attention in the last few decades given the major contributions in numerous fields such as coatings, catalysis, sensors, and electronics [1,2,3]. In terms of fabrication, one straightforward approach is the use of organic templates, including carbon nanotubes [4,5], polymers [6,7], or even biological objects such as viruses [8,9], on which an inorganic matrix is deposited in situ. To preserve the delicate template structure, mild deposition conditions are necessary. Next to the frequently used sol–gel procedure [6,10,11], chemical bath deposition (CBD) has been proven beneficial, in particular for the synthesis of nanostructured hybrid materials with a finely tuned thickness of the deposited inorganic phase on the organic template. In CBD, an (organic) template is mineralized while being immersed in a precursor solution at moderate temperatures, typically below 100 °C [12]. Additives such as polymers [13] or small molecules like amino acids [14] may be present and used as a structure-directing agent to precisely control the growth of the solid with respect to size, morphology, and crystallinity. Such control is achieved by the ability of the additives to stabilize the dissolved ions, direct and restrict the particle growth, and prevent nanoparticle agglomeration by steric and/or electrostatic interactions. Using this approach, dense and smooth thin films and nanostructured hybrid materials of metal chalcogenides like CdS [15,16,17], TiO_2_ [18,19,20], and ZnO [21,22,23] were fabricated through organic templates. Among them, the transparent semiconductor ZnO is of particular interest as it covers a vast range of applications from optoelectronics [24,25] to gas sensors [26,27] and lasing devices [28,29], etc.

Applying a well-established CBD route and using various organic templates such as self-assembled monolayers [30,31,32,33], polymers [32,34], polymer foams [35], DNA [36], and viruses [37,38,39,40], ZnO hybrid materials were prepared from a methanolic ZnO precursor solution in the presence of polyvinylpyrrolidone (PVP). In addition, close-packed assemblies of polymer nanoparticles were mineralized by applying the same approach, followed by the removal of the particles to produce ZnO inverse opals [41,42]. They are a promising candidate for photonic applications [43,44] or as catalyst solid supports in heterogeneous catalysis [45], combining the ordered porous structure with the favorable electrical and optical properties of ZnO. First insights into the role of PVP on the deposition rate, mechanism, and film morphology of ZnO films obtained applying this CBD approach were given in the studies of Lipowsky et al. [30,31,33]. It was found that in the absence of PVP, the immediate precipitation of micron-sized ZnO particles takes place, whereas intermediate PVP volume fractions (0.06 < *ϕ*_PVP_ < 0.14) are required for the successful preparation of thin, homogeneous nanocrystalline ZnO films. It was also shown that the deposition of ZnO occurs via a transient amorphous phase in which needle-like particles containing both zinc and PVP are formed [33], similar to the well-known formation of intermediate amorphous phases in biomineralization and bioinspired mineralization processes [46,47].

A powerful technique for the in situ study of the structural evolution during nanoparticle synthesis is time-resolved small-angle X-ray scattering (SAXS), which can be complemented by ex situ imaging techniques such as transmission electron microscopy (TEM). As far as ZnO formation via the sol–gel method is concerned, Caetano et al. investigated the nucleation and growth of ZnO nanoparticles synthesized through an additive-free sol–gel process via time-resolved tandem SAXS/UV-Vis and X-ray adsorption fine structure (XAFS) experiments [48,49,50]. These studies revealed that ZnO nuclei formed by supersaturation-induced nucleation are subject to the so-called oriented attachment growth [51,52], where particles aggregate by coalescence. In the late stages, Ostwald ripening [53,54] with dissolution and reprecipitation processes dominates. Analogies were found by Herbst et al. during the thermolysis of Zn-oleate precursors, verified by in situ SAXS/WAXS/UV-Vis measurements [55]. In their study, a classical homogeneous nucleation and growth model was applied to describe the process of ZnO nanoparticle synthesis. A nonclassical growth behavior where both diffusion- and surface-control occur was observed when the PVP-mediated solvothermal synthesis of ZnO nanorods was studied by ex situ SAXS and TEM [56]. 

In this study, we use time-resolved USAXS and SAXS for the in situ investigation of the role of the organic additive PVP during the formation and growth of individual ZnO nanoparticles and ZnO clusters in a methanolic precursor solution applying the above-mentioned CBD method. As found in previous studies, this method requires a relatively high volume fraction of PVP (*ϕ*_PVP_ = 7.1 × 10^−2^); therefore, its scattering contribution must be considered when analyzing the scattering data. Accordingly, the first part of this work deals with the study of the polymer structure in the precursor solution before initiating the synthesis of ZnO particles by heating. In the second part, we clarified the ZnO particle formation performed at two temperatures, i.e., 58 °C and 63 °C, via time-resolved synchrotron USAXS measurements. Form factor modeling and model-independent invariant analyses of the recorded USAXS data allowed us to not only describe the data quantitatively but also to unravel the ZnO nanoparticle formation mechanism.

## 2. Materials and Methods

### 2.1. Synthesis of ZnO Nanoparticles

ZnO nanoparticles were synthesized following an established procedure [35,41,42]. Briefly, three methanolic stock solutions of zinc acetate dihydrate (ZnAc_2_ × 2 H_2_O) (37.4 mM, Sigma-Aldrich, St. Louis, MO, USA), tetraethylammonium hydroxide (TEAOH) (79.0 mM, Sigma-Aldrich), and polyvinylpyrrolidone (PVP) (25.7 mM) (Sigma-Aldrich, *M*_w_ = 10,000 g/mol (10 k), Lot # BCBJ4889V) were used to prepare the precursor solution. While the stock solutions can be stored for a longer period of time, the precursor solution was prepared freshly before use to avoid unwanted ZnO formation. Equal volumes of the ZnAc_2_ and PVP stock solution were mixed, followed by dropwise addition (1 mL/min) of the same volume of the TEAOH stock solution under continuous stirring. Thus, a precursor solution with a volume ratio of 1:1:1 of the stock solutions and final concentrations of [PVP] = 8.6 mM, [Zn^2+^] = 12.5 mM, and [TEAOH] = 26.3 mM was obtained. For the synthesis of ZnO particles, the precursor solution was heated to *T* = 58 °C and 63 °C, respectively.

### 2.2. Characterization

#### 2.2.1. In-House Small-Angle X-ray Scattering

Room temperature studies of the precursor solution were performed on a SAXSess instrument from Anton Paar (Graz, Austria). The instrument was equipped with a Cu K_α_ X-ray source (*λ* = 1.54 Å) operated in line collimation mode. Samples were filled into a quartz capillary flow cell and data were collected at 25 °C using imaging plates from Fujifilm (Greenwood, SC, USA), which were read by a cyclone scanner from PerkinElmer (Covina, CA, USA). One-dimensional corrected and reduced scattering profiles *I*(*q*) as a function of the absolute value of the scattering vector *q*, with *q* = 4π/*λ* sin(*θ/2*), where *θ* is the scattering angle, were obtained using Anton Paar’s SAXSquant 2D and 1D software, including the subtraction of the dark current and the empty cell scattering. To normalize the data to an absolute scale, water was used as a secondary calibration standard [57]. The scattering data were analyzed using SasView version 5.0.4, with slit-smearing accounted for in the fit model. Alternatively, the slit-smeared data were desmeared using SAXSquant 2.0.

#### 2.2.2. Ultra-Small- and Small-Angle Synchrotron X-ray Scattering

USAXS and SAXS data were collected at the 9-ID-C beamline at the Advanced Photon Source at Argonne National Laboratory (Argonne, IL, USA) [58,59]. A combined *q* range between 3.7 × 10^−4^ Å^−1^ and 1.7 Å^−1^ was covered using an X-ray energy of 21 keV (*λ* = 0.5895 Å) and an X-ray flux of about 5 × 10^12^ photons mm^−2^ s^−1^. To study the time-dependent growth of the ZnO particles, the precursor solution was filled into an NMR tube (Wilmad-LabGlass^TM^, WG-1000-4, inner diameter 4 mm), which was placed in a thermostated home-built cell holder. USAXS scattering data were recorded starting at *T* = 25 °C, followed by rapid heating (1 K/s) to *T* = 58 °C and 63 °C, respectively, with collection times of 100 s each. Before and after the time-resolved USAXS measurements, SAXS patterns were acquired with collection times of 20 s.

The slit-smeared USAXS data were reduced for instrumental background and empty cell scattering, normalized to absolute scale, and desmeared using the instrument data reduction software package Indra for Igor Pro 9.0. For the SAXS data and to combine the USAXS and SAXS data, Nika [60] and Irena [61] were used, respectively. The scattering data were analyzed using SasView version 5.0.4, with the slit-smearing accounted for in the fit model.

#### 2.2.3. Transmission Electron Microscopy

For particle visualization, TEM images were collected on a EM10 from Zeiss (Jena, Germany) operated at 60 kV. Each nanoparticle solution was diluted in methanol at room temperature. A small drop of the diluted sample was placed on a TEM copper grid covered with a thin holey carbon film. The drop was then gently blotted to remove the excess sample. After the solvent had completely evaporated, the dry grid containing the particles was transferred to the microscope. A 1 k × 1 k CCD camera was used to record the TEM images, which were analyzed using the software *ImageJ* to evaluate the size of at least 100 ZnO particles and clusters, respectively, based on which the mean radii *R*_ZnO,i,TEM_ and the standard deviations σ_i,TEM_ were determined using a Gaussian size distribution function.

## 3. Results and Discussion

In the following, we first describe the structural investigation of PVP in the methanolic ZnO precursor solution by in-house SAXS and USAXS/SAXS. We then discuss the time-resolved synchrotron USAXS measurements performed to determine the kinetic processes of ZnO particle formation and growth in situ. We show that a quantitative analysis of all scattering data is possible when considering the scattering contributions of the previously determined stabilizing polymer structure and two ZnO species, i.e., ZnO nanoparticles and their clusters. In the last part, we use the determined time evolution of the size and volume fraction of ZnO particle clusters to study the kinetics using the two-step Finke–Watzky model [62,63,64,65].

### 3.1. PVP Structure in the Precursor Solution

The chemical bath deposition (CBD) of ZnO nanoparticles studied in this work is based on the thermally induced hydrolysis of the zinc precursor zinc acetate (ZnAc_2_) in a methanolic solution under basic conditions adjusted by the organic base tetraethylammonium hydroxide (TEAOH). Furthermore, the ion-stabilizing and structure-directing agent PVP (10 k) is added at a volume fraction of *ϕ*_PVP_ = 7.1 × 10^−2^. As a prerequisite for the comprehensive investigation of the ZnO particle formation by USAXS/SAXS, the contribution of all components to the total scattering should be clarified first. Therefore, we started with the investigation of the PVP structure in methanol before and after the addition of the respective components as well as the PVP structure in the final composition of the precursor solution by SAXS at 25 °C.

**Figure 1:**
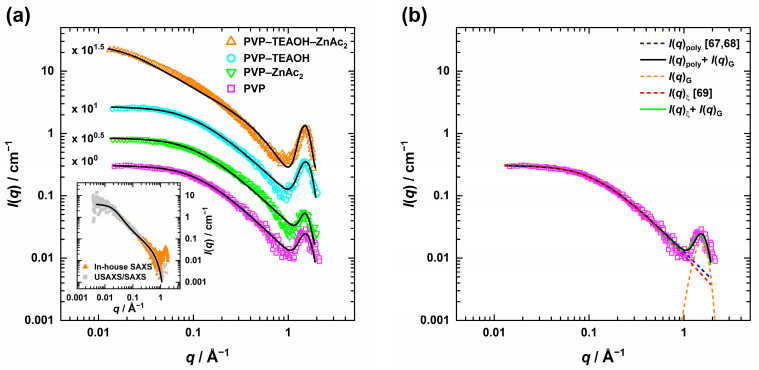
(**a**) Slit-smeared SAXS profiles of PVP at *ϕ*_PVP_ = 7.1 × 10^−2^ in methanol with the respective compounds and the final composition (TEAOH = 26.3 mM, ZnAc_2_ = 12.5 mM), measured at *T* = 25 °C. The scattering curves were described using the polymers with excluded volume effects model [67,68], and for the final composition, the flexible cylinder model [70,71] combined with a Gaussian, respectively (solid lines). To help the reader distinguish between the scattering curves, they are displaced by an arbitrary factor. Inset: Comparison of desmeared in-house SAXS and USAXS/SAXS data for the final composition with the flexible cylinder fit. (**b**) Applied fit models (Equations (3) and (4)) and their respective scattering contributions are shown for PVP in methanol.

In Figure 1a, the recorded slit-smeared SAXS intensities *I*(*q*) were plotted as a function of the scattering vector *q* using a double-logarithmic representation. The scattering curve of PVP in pure methanol *ϕ*_PVP_ = 7.1 × 10^−2^ (8.6 mM) (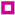) shows a Guinier region at low *q* with almost constant scattering intensity. As *q* increases, a power law decay is observed until a minimum is reached at *q* ≈ 1 Å^−1^, followed by the intermolecular C-C chain interaction peak [66] at *q* ≈ 1.5 Å^−1^. Similar behavior was found when 12.5 mM ZnAc_2_ (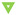) or 26.3 mM TEAOH (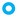) were added while keeping the polymer amount constant at *ϕ*_PVP_ = 7.1 × 10^−2^. However, for the final composition of the precursor solution (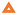), instead of a Guinier region for *q* < 0.1 Å^−1^, a power law decay with *I*(*q*) ≈ *q*^−1.4^ can be seen. Thus, the presence of both ZnAc_2_ and TEAOH results in a significantly different structure of the polymer.

In general, the recorded scattering curves provide information about the polymer structure on different-length scales: the Guinier region gives the overall dimension of the polymer, i.e., its radius of gyration *R*_g_(PVP), while the exponent *m* of the power law decay is related to the polymer–solvent interactions [67,68]. Furthermore, the correlation length ξ, quantifying the end-to-end distance of a coil or the mesh size, i.e., the mean diameter of a cavity within the polymer network [69], respectively, can be extracted.

To determine *R*_g_(PVP) and the exponent *m* of the power law decay, the scattering curves, except for the final composition, were analyzed according to the polymers with excluded volume effects model [67,68]:

(1) $${I\left(q\right)}_{poly}=I_{0}\left[\frac{m}{U^{\frac{m}{2}}}\gamma \left(\frac{m}{2},\;U\right)-\frac{m}{U^{m}}\gamma \left(m,\;U\right)\right],$$

where *I*_0_ corresponds to the forward scattering intensity, γ is the incomplete gamma function, and the parameter U is defined by

(2) $$U=\frac{q^{2}{R_{g}}^{2}(2/m+1)(2/m+2)}{6}.$$

Since *I*(*q*)_poly_ does not account for large *q* features such as the intermolecular C-C chain interaction peak [66], Equation (1) was combined with a Gaussian *I*(*q*)_G_, which finally leads to the following expression:

(3) $$I\left(q\right)={I\left(q\right)}_{poly}+{I(q)}_{G}={I\left(q\right)}_{poly}+G\cdot \exp\left(\frac{1}{2}{\left(q-q_{G}\right)}^{2}/{\sigma_{G}}^{2}\right).$$

Herein, *G* is the scaling factor for the Gaussian with the maximum peak position at *q*_G_, and the peak width σ_G_. Note that the solid lines in Figure 1a correspond to the final fit following Equation (3), while in Figure 1b, the final fit as well as the respective contributions of Equation (3) are shown exemplarily for the scattering pattern of PVP in methanol (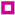).

The correlation length ξ was obtained, separately, by applying the correlation length model [69] *I*(*q*)_ξ_, which was again combined with a Gaussian:

(4) $$I\left(q\right)={I\left(q\right)}_{\xi }+{I(q)}_{G}=\frac{A}{q^{m’}}+\frac{C}{1+{\left(q\xi \right)}^{b}}\;+{I(q)}_{G}$$

where the first term accounts for the scattering of large-scale polymer clusters, and the second term for the polymer chains. *A* and *C* are scaling parameters and *m*′ and *b* are the exponent of the power law decay(′ denotes for small *q*′s) and the Lorentzian exponent, respectively. In cases where the low *q* power law decay was absent, the first term was neglected (*A* = 0). Also, for Equation (4), the respective contributions, together with the final fit, are shown exemplarily for PVP in methanol (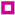) in Figure 1b. 

Applying Equations (3) and (4) and taking the slit-smearing into account, it was possible to describe the scattering curves quantitatively. The best-fit parameters are compiled in Tables S1 and S2 in the Supplementary Materials, while the corresponding Guinier radius *R*_g_(PVP), the exponent *m* of the power law decay at high *q*′*s*, and the correlation length ξ_PVP_ are summarized in Table 1. Following the analysis of the scattering data of PVP in methanol (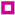) with Equation (3) revealed a high *q* power law decay exponent of *m* ≈ 1.5, suggesting that the polymer chains are no longer in an ideal (*m* = 2) or swollen (*m* = 5/3) state but already approach a rod-like state (*m* = 1) [68,72]. A similar behavior was found by Sapir et al. [73] once the overlap concentration c* of PVP-10k in D_2_O (*ϕ*_PVP_ ~ 4.8 × 10^−2^) was exceeded and the semidilute polymer regime began. This regime refers to a state in which the polymer forms a network with overlapping and entangled chains. When PVP was studied at lower volume fractions (*ϕ*_PVP_ = 1.0, 2.0, and 4.0 × 10^−2^) in methanol, shown in the Supplementary Materials Figures S1 and S2 and Tables S3 and S4, ideal chain behavior was observed exclusively for the lowest PVP volume fraction *ϕ*_PVP_ = 1.0 × 10^−2^. At *ϕ*_PVP_ = 2.0 × 10^−2^, the exponent of the power law decay drops steeply and then slowly decreases with increasing PVP amount, while *R*_g_(PVP) decreases continuously. Furthermore, the change of the scaling dependency of the correlation length, observed between *ϕ*_PVP_ = 4.0 and 7.1 × 10^−2^, confirms that the PVP concentration in the methanolic precursor solution can be assigned to the semidilute regime [74].

The analysis of the scattering data from the methanolic PVP solution (*ϕ*_PVP_ = 7.1 × 10^−2^) in which ZnAc_2_ (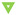) or TEAOH (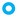) was added revealed a slight increase in *R*_g_(PVP) (see Table 1) while the decrease in the exponent of the power law decayindicates that the polymer becomes more rod-like [72]. Though PVP is a nonionic polymer, the pyrrolidone head group is polar and interacts with the ions of the ZnO precursor and the organic base [33,75]. Their accumulation along the polymer chains, leading to repulsion of the segments within the polymer chain, can explain why the polymer chain becomes slightly stiffer and more swollen compared to the ion-free solution. This effect is slightly more pronounced with TEAOH, whose concentration is more than twice of ZnAc_2_.

**Table 1:** Parameters obtained from the analysis of SAXS data from PVP in methanol at *ϕ*_PVP_ = 7.1 × 10^−2^ before and after adding 12.5 mM ZnAc_2_ and 26.3 mM TEAOH, respectively, as well as the final composition. While the polymer with excluded volume model [67,68] provided the radius of gyration *R*_g_(PVP) and the high *q* power law exponent *m,* the exponent of the power law decay at low *q m*′ and the correlation length ξ_PVP_ were obtained through the correlation length model [69].

Sample	*R*_g_(PVP)/nm	*m*	*m*′	ξ_PVP_/nm
PVP	1.5 ± 0.1	1.52 ± 0.01	-	1.1 ± 0.1
PVP–ZnAc_2_	1.7 ± 0.1	1.46 ± 0.01	-	1.1 ± 0.1
PVP–TEAOH	1.8 ± 0.1	1.45 ± 0.01	-	1.3 ± 0.1
PVP–TEAOH–ZnAc_2_	6.3 ± 0.3 ^a^	-	1.55 ± 0.01	2.9 ± 0.2

^a^ obtained by the Guinier analysis.

Discussing the scattering data of the precursor solution (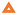) (where both ZnAc_2_ and TEAOH are present) in more detail, the power law decay (*I*(*q*) ≈ *q*^−1.4^) recorded in the low *q* limit of the in-house SAXS instrument provides little information on the global structure and size of the polymer. This information can be obtained by extending the *q*-range towards much smaller values using the USAXS/SAXS setup at the 9-ID-C beamline at the Advanced Photon Source at the Argonne National Laboratory [58,59]. The inset in Figure 1 shows a good agreement between the desmeared in-house (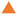) and USAXS/SAXS (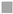) data. In addition, the combination of the data clearly shows the transition of the power law into a Guinier region at *q* < 0.01 Å^−1^. Utilizing Guinier’s approximation for low *q* (ln *I*(*q*) ≈ ln *I*_0_ − (*R*_g_^2^/3) *q*^2^), we obtained *R*_g_(PVP) = 6.3 ± 0.3 nm, which corresponds to a fourfold increase compared to *R*_g_(PVP) in pure methanol and in solutions containing only ZnAc_2_ or TEAOH. This significant change in the PVP structure caused by the increased ion concentration due to the presence of both ZnO precursor and base is most likely related to a stronger repulsion of the segments within the polymer chain, leading to a further increase in the chain stiffness. This hypothesis is supported by the increase in mesh size determined from the analysis at medium *q*’s to ξ_PVP_ = 2.9 ± 0.2 nm. Moreover, ZnAc_2_ might start to hydrolyze even at 25 °C, changing its chain flexibility so that the first particle nuclei could have formed within the PVP. However, this reaction is significantly accelerated only by increasing the temperature. 

For the quantitative analysis of the precursor solution data, we used the flexible cylinder model for single semiflexible polymer chains [70,71], which has already been applied to the scattering of aqueous PVP-40 k and -360 k solutions [76], combined with a Gaussian to describe the intermolecular C-C chain interaction peak [66] at high *q*. Accordingly, the scattering intensity was modeled by

(5) $$I\left(q\right)={I\left(q\right)}_{flexC}+{I(q)}_{G}=\phi_{PVP}∆\rho^{2}V_{C}S\left(q,L,b\right)P\left(q,R_{C}\right)+{I(q)}_{G}$$

where *ϕ*_PVP_ is the volume fraction of PVP, ∆*ρ*^2^ is the square of the scattering length density difference between PVP (*ρ*_PVP_ = 11.0 × 10^−6^ Å^−2^) and methanol (*ρ*_MeOH_ = 7.5 × 10^−6^ Å^−2^), *V*_C_ is the volume of the cylinder and *S*(*q*, *L*, *b*) is the scattering of a polymer chain with excluded volume effects with the contour length *L*, the Kuhn length *b,* a measure of the local stiffness, and the circular cross section *P*(*q*, *R*_C_) of a cylinder with the radius *R*_C_. The comprehensive mathematical expression of *S*(*q*, *L*, *b*) can be found in the work of Chen et al. [71], while *P*(*q*, *R*_C_) is given by the first-order Bessel function *J*_1_ with $P\left(q,R_{C}\right)={\left(\frac{2J_{1}\left(qR_{C}\right)}{qR_{C}}\right)}^{2}$.

As can be seen in Figure 1, this model is able to describe the smeared and desmeared (inset) scattering data of the precursor solution, almost quantitatively, yielding a contour length of *L* = 80 ± 1 nm, a Kuhn length of *b* = 3.3 ± 0.3 nm, and a cross-section radius of *R*_C_ = 0.27 ± 0.01 nm. *b* and *R*_C_ are in the same range, as already reported for PVP-40 k and -360 k in dilute aqueous solutions (*b* = 3.0 ± 0.2 nm and *R*_C_ ≈ 0.1 nm [76]) as well as for PVP-2 k to -2200 k in 0.1 M aqueous NaAc solution (*b* ≈ 1.7 − 2.4 nm and *R*_C_ = 0.25 ± 0.01 nm [77]). Estimating the radius of gyration from the contour and Kuhn length with *R_g_*^2^ ≈ *Lb*/6 [78], we obtain *R*_g_(PVP) = 6.5 ± 0.4 nm, in good agreement with the Guinier analysis. Note that in the region around *q* ~ 0.1 Å, the modeled scattering curve shows a stronger curvature than the recorded data. This small but systematic difference is most likely related to polymer–polymer interactions, which are neglected in the applied model [70].

The shape- and size-independent invariant analysis of the desmeared USAXS/SAXS data (*q* < 1 Å^−1^), which are on absolute scale, has shown that the scattering length density difference ∆*ρ* had to be increased from ∆*ρ* = *ρ*_PVP_ − *ρ*_MeOH_ = 3.5 × 10^−6^ Å^−2^ to ∆*ρ* = 5.6 × 10^−6^ Å^−2^ in order to obtain the experimental PVP volume fraction. The same increase in contrast factor was also obtained from the analysis of desmeared in-house SAXS data as well as of additional SAXS data recorded at the ID02 beamline at the European Synchrotron Radiation Facility (ESRF) in Grenoble, France [79]. The higher X-ray scattering contrast might be related to ion association along the polymer chain as well as additional specific volume effects, which both increase the scattering length density of the polymer.

In summary, due to the relatively high volume fraction of PVP, the initial state of the precursor solution is characterized by the scattering of a PVP network. Ions are distributed along and within the cavities of the PVP network, where they interact with the pyrrolidone moiety of the polymer, leading to a semiflexible polymer chain behavior.

### 3.2. In Situ Study of ZnO Particle Formation and Growth

**Figure 2:**
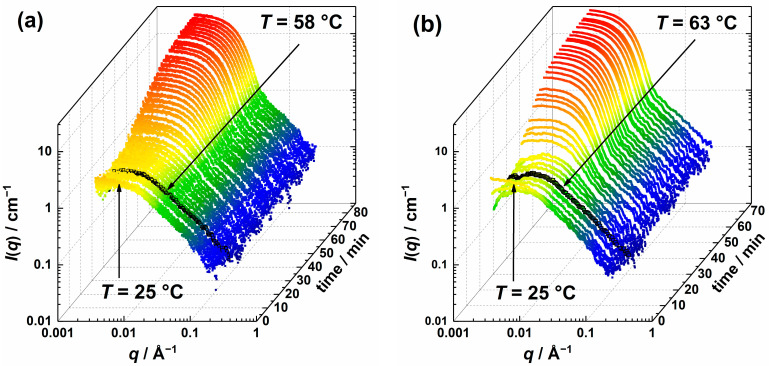
Scattering intensity colored stack-plot of slit-smeared USAXS curves during the ZnO particle formation at *T* = 58 °C (**a**) and *T* = 63 °C (**b**). The initial scattering curve at *T* = 25 °C and the curve at which the target temperature was reached are marked with arrows.

Having studied the initial state of the precursor solution at 25 °C, we then focused on elucidating the formation of ZnO particles induced by heating. Typically, the reaction is carried out at 60 °C, as ZnAc_2_ hydrolyzation and mass transport during the ZnO formation are accelerated at this temperature. Herein we chose to study the ZnO kinetics at *T* = 58 °C and 63 °C. The reason for that is the significantly slower deposition below 50 °C, as confirmed in preliminary studies, while the upper limitation for the reaction temperature is given by the boiling point of methanol at 65 °C. Figure 2 shows the time-dependent slit-smeared USAXS curves starting from 25 °C (*t* = 0 min) followed by rapid heating to *T* = 58 °C (a) or 63 °C (b).

During heating and temperature equilibration the precursor solution to *T* = 58 °C (Figure 2a), which took about 10 min, the scattering curves are dominated by the scattering of the polymer network with the above-mentioned features of a Guinier region at low *q*, followed by a power law decay. The slight decrease in the intensity at low *q* might relate to the heating with an inhomogeneous temperature distribution inside the solution. Potential over subtraction of solvent scattering, which was measured at *T* = 58 °C, might be another explanation for this decrease.

Subsequently, for reaction times *t* > 13 min, the scattering intensities at low *q* increase continuously, which can be attributed to the form factor contribution of newly formed ZnO species, while above *q* ≥ 0.1 Å^−1^, the scattering is dominated by the PVP scattering. As the reaction progresses, the form factor minimum becomes increasingly apparent and shifts to lower *q* values, suggesting the growth of the ZnO species. Finally, the increase in the low *q* scattering intensity levels off, indicating that the precursor ZnAc_2_ has been consumed; therefore, the data acquisition was stopped after 87 min. Contrary to what Lipowsky et al. [33] have seen when ZnO films are formed in the presence of PVP via CBD, no additional contribution of needle-like species with a length scale between 20 and 50 nm was observed in the scattering curves.

A similar behavior of the scattering curves can be seen during the formation of ZnO particles at *T* = 63 °C in Figure 2b. Compared to the formation at 58 °C, the decrease in the forward scattering observed in the curves recorded at the early stages of the process is even more pronounced, most likely due to over subtraction of the background, measured at the higher final temperature. The earlier appearance of the form factor signature and the earlier leveling off of the increase in forward scattering after 75 min clearly indicate that the reaction is faster at higher temperatures. This can be explained by the enhanced mass transport with increasing temperature, which accelerates the formation of ZnO.

**Figure 3:**
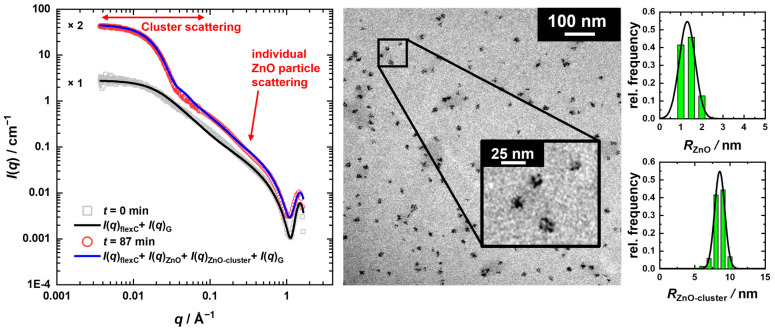
Left: Slit-smeared USAXS/SAXS curves measured before heating (*t* = 0 min, *T* = 25 °C) and after the reaction was stopped (*t* = 87 min, *T* = 58 °C), displaced by an arbitrary factor. To model the final scattering curve, the flexible cylinder model [70,71] used for *t* = 0 min was additively combined with two polydisperse spherical form factors [80] for the scattering contributions of the ZnO particles and their clusters, as well as with a Gaussian. Right: TEM image taken after the ZnO synthesis at *T* = 58 °C with the corresponding volume-weighted size distribution of the ZnO particles and clusters, described by a Gaussian size distribution, respectively.

A comparison of the first (*t* = 0 min, *T* = 25 °C) and the last (*t* = 87 min, *T* = 58 °C) time-resolved USAXS/SAXS scattering curves are shown by means of the ZnO particles’ formation at *T* = 58 °C (Figure 3, left). This comparison highlights the presence of the additional form factor contribution (*q* ≤ 0.08 Å^−1^) in the scattering curve recorded at the process end (*t* = 87 min). A closer look also reveals a weak shoulder around *q*~0.15 Å^−1^. One exemplary image of complementary TEM studies taken from a separately prepared solution after an analogous heating procedure starting from 25 °C to 58 °C is shown in the center of Figure 3. Therein, two species are visible: smaller ones with a radius of *R*_ZnO,TEM_ = 1.3 ± 0.7 nm as well as agglomerates of those with an average radius of *R*_ZnO-cluster,TEM_ = 8.5 ± 1.4 nm. We will refer to the smaller particles as individual ZnO particles and to the larger ones as clusters of ZnO particles. Strikingly, the diameter of the individual ZnO particles is only slightly smaller than the mesh size within the PVP network (ξ_PVP_ ≈ 2.9 nm), indicating initial growth of the ZnO particles in the network cavities before cluster formation occurs. Furthermore, the average cluster radius is only slightly larger than the Guinier radius of PVP in the precursor solution with *R_g_*(PVP) = 6.3 ± 0.3 nm.

Comparing the final cluster radius obtained from the USAXS/SAXS scattering curve analysis (*R*_ZnO-cluster,f_ = 10.5 ± 1.9 nm) to TEM, one may assume that the clustered particles are surrounded by a PVP layer that is not visible in the TEM, similar to what has been demonstrated for silica-polymethyl methacrylate (PMMA) nanocomposites by SANS [81]. Since we were not able to study such a layer independently with SANS, we used a simple approach for the quantitative analysis of the time-resolved USAXS/SAXS data. In this approach, the scattering contribution of the PVP network described by the flexible cylinder model [70,71] *I*(*q*)_flexC_ was additively combined with the scattering contributions of the individual ZnO particles *I*(*q*)_ZnO_ and their clusters *I*(*q*)_ZnO-cluster_, each modeled by the form factor of polydisperse spheres [80], as well as a Gaussian *I*(*q*)_G_ for the intermolecular C-C chain interaction peak [66] at high *q*. Thus, the total intensity is given by

(6) $$I\left(q\right)=\left.I{\left(q\right)}_{flexC}+{I\left(q\right)}_{ZnO}+{I\left(q\right)}_{ZnO-cluster}+{I\left(q\right)}_{G}+I_{background}\right.$$

where

(7) $${I\left(q\right)}_{ZnO,i}=\phi_{ZnO,i}V_{ZnO,i}∆\rho^{2}\int P\left(q,R_{ZnO,i}\right)f\left(R_{ZnO,i},R_{ZnO,i,f},\;\sigma_{i}\right)dR$$

with *ϕ*_ZnO,i_ and *V*_ZnO,i_ being the volume fractions and average volumes of particles and particle clusters, respectively. $∆\rho^{2}$ is the scattering length density difference between ZnO (*ρ*_ZnO_ = 44.8 × 10^−6^ Å^−2^) and methanol (*ρ*_MeOH_ = 7.5 × 10^−6^ Å^−2^) and *P*(*q*,*R*_ZnO,i_) = ${\left(\frac{3J_{1}\left(qR_{ZnO,i}\right)}{qR_{ZnO,i}}\right)}^{2}$ the form factor of a sphere with the radius *R*_ZnO,i_. To account for size distribution effects, the form factor was convoluted with a Gaussian size distribution functions *f*(*R*_ZnO,i_, *R*_ZnO,i,f_, σ_i_) which yields the mean radius *R*_ZnO,i,f_ and the width σ_i_.

Using this approach, we were able to describe the recorded time-resolved scattering curves almost quantitatively, as shown in the left panel of Figure 3. In this regard, the solid line for *t* = 0 min corresponds to the final fit following Equation (5), with a PVP chain contour length of *L* = 80 ± 1 nm, a Kuhn length of *b* = 3.3 ± 0.3 nm, and a cross-section radius of *R*_C_ = 0.27 ± 0.01 nm. For the solid line at *t* = 87 min (*T* = 58 °C), Equation (6) was used. Here, the previously determined characteristics of the PVP chains with a slightly adjusted Kuhn length of *b* = 4.0 ± 0.3 nm were applied, while the polydispersity *p*_i_ = σ_i_/*R* _ZnO,i0_ = 0.18 of both the ZnO particles and clusters was kept constant. Hence, the analysis of the USAXS/SAXS curve measured after the termination of the reaction yields average radii of *R*_ZnO,f_ = 1.5 ± 0.3 nm and *R*_ZnO-cluster,f_ = 10.5 ± 1.9 nm for the individual ZnO particles and their clusters, respectively. While the size of the individual ZnO particles matches that from the TEM images within the measurement error, the size of the clusters is slightly larger, most probably due to a surrounding PVP layer, as discussed before. The respective contributions of Equation (6) to the final fit are shown in Figure S3 in the Supplementary Materials.

Also, for the ZnO formation performed at *T* = 63 °C, starting from *T* = 25 °C, the final ZnO particle and cluster size obtained from TEM images (*R*_ZnO,TEM_ = 1.7 ± 0.5 nm, *R*_ZnO-cluster,TEM_ = 9.7 ± 1.9 nm), shown exemplarily in Figure S4 in the Supplementary Materials, are in good accordance with that from USAXS/SAXS (*R*_ZnO,f_ = 1.5 ± 0.3 nm, *R*_ZnO-cluster,f_ = 11.8 ± 2.1 nm). Comparing the size of the clusters obtained from CBD at the two different temperatures, the temperature increase leads to slightly larger ZnO clusters, which is related to accelerated kinetics at higher temperatures. Hereby, a higher number of individual ZnO particles might be formed, which are subsequently consumed faster by the formation of the clusters.

**Figure 4:**
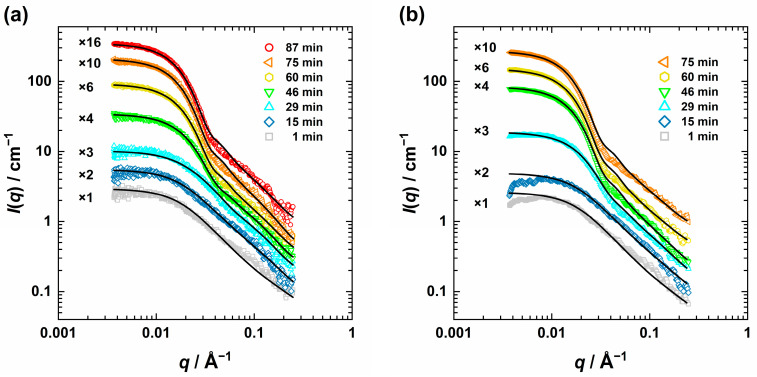
Selected slit-smeared USAXS curves measured during the ZnO particle formation at *T* = 58 °C (**a**) and *T* = 63 °C (**b**) at different times described by a combination of the flexible cylinder model [70,71] with two sphere form factor contributions [80].

Figure 4 shows selected time-resolved USAXS scattering curves recorded at *T* = 58 °C (Figure 4a) and *T* = 63 °C (Figure 4b), which are almost quantitatively described by the combination of the flexible cylinder model [70,71] and the two spherical form factor contributions [80]. It should be noted that for *t* < 13 min, we could not obtain any information about the ZnO formation due to the dominance of the PVP scattering contribution.

**Figure 5:**
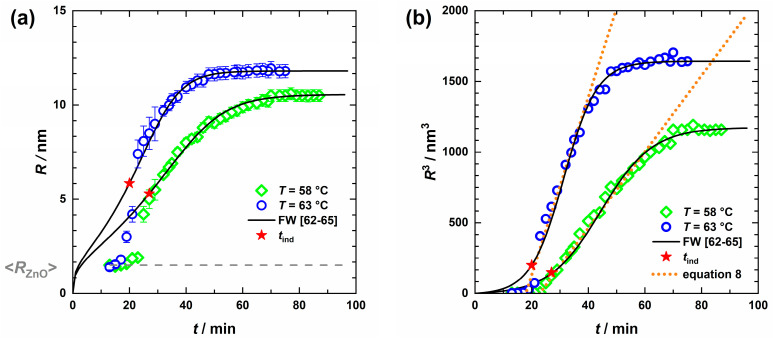
Time evolution of ZnO cluster radii at *T* = 58 °C (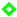) and *T* = 63 °C (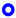) with ZnO nanoparticles (dashed line) held constant (**a**) obtained from the USAXS curve analysis, as well as the corresponding cube of radii (**b**). A phenomenological approach can describe the growth period (dotted lines), yet to fit the nucleation and growth phase the Finke–Watzky model [62,63,64,65] (solid lines) is more suitable and also provides the induction time *t*_ind_ (red stars).

**Figure 6:**
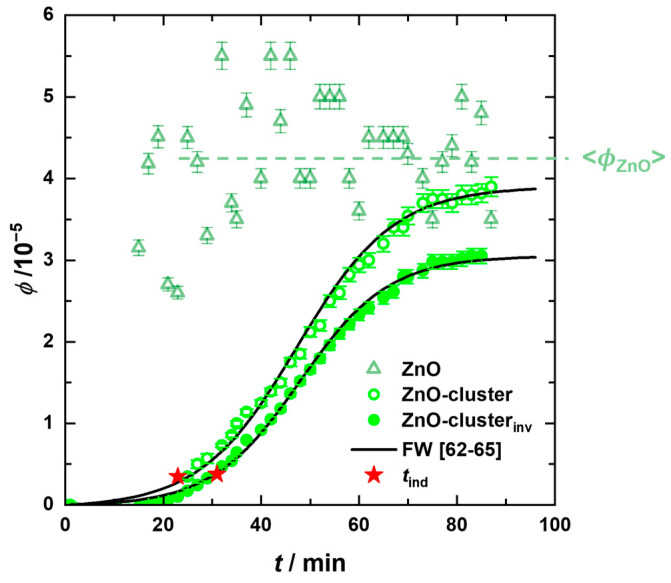
Volume fractions of the ZnO nanoparticles *ϕ*_ZnO_(*t*)*_I_*_(*q*)_ and the clusters *ϕ*_ZnO-cluster_(*t*)*_I_*_(*q*)_ determined from the fitted USAXS curves at *T* = 58 °C, together with the cluster volume fractions obtained from the invariant analysis *ϕ*_ZnO-cluster,inv_(*t*) as a function of time. The solid lines correspond to the Finke–Watzky modelling [62,63,64,65] which also yields the induction time *t*_ind_ (red stars).

The results of the quantitative analysis of the scattering data according to Equation (6), with the free fitting parameters being the mean radii from the ZnO particles, *R*_ZnO,f_, and that from the clusters *R*_ZnO,cluster,f_ as well as their volume fractions *ϕ*_ZnO_ and *ϕ*_ZnO-cluster_, are shown in Figure 5 and Figure 6. While Figure 5 shows the radius (*R*) and *R*^3^(*t*) of the ZnO nanoparticles and ZnO clusters as a function of time for *T* = 58 °C and *T* = 63 °C, the time evolution of the respective volume fraction for *T* = 58 °C is shown in Figure 6.

As can be seen in Figure 5a, the size of the formed ZnO species for *T* = 58 °C remains almost constant during a short period (~between *t* = 13 and 18 min). Thereafter, cluster growth occurs, where the average size of these clusters initially increases rapidly before slowly approaching the final size due to the consumption of zinc precursor. Performing the reaction at *T* = 63 °C, a similar but steeper growth profile was obtained, during which the clusters are consistently larger. For comparison, the ZnO particle size appeared to remain constant and was kept fixed at *R*_ZnO,f_ = 1.5 ± 0.3 nm (dashed line in Figure 5a).

Interestingly, when plotting *R*^3^_ZnO-cluster,f_ as a function of time, as shown in Figure 5b, a linear increase starting from *t*~20 min is observed, which levels off to a plateau. The former region can be associated with the growth phase of the ZnO clusters by agglomeration of individual ZnO particles. Thus, a reasonable description of the growth phase is given by

(8) $${R\left(t\right)}^{3}=k(t_{cluster})+{R_{ZnO,f}}^{3}$$

with *k* being the temperature-dependent growth rate constant, and *t*_cluster_ being the time after which clusters start to grow *t*_cluster_ = *t* − *t*_cluster,0_ and *R*_ZnO,f_ the individual ZnO nanoparticle radius. The analysis of *R*^3^_ZnO-cluster,f_(*t*) with Equation (8) (dotted lines in Figure 5b) yielded *k*(*T* = 58 °C) = 0.44 ± 0.01 nm^3^/s with *t*_cluster,0_ (*T* = 58 °C) = 23 min and *k*(*T* = 63 °C) = 1.05 ± 0.01 nm^3^/s with *t*_cluster,0_ (*T* = 63 °C) = 18 min. For comparison, much smaller rate constants in the order of ~10^−3^ nm^3^/s were found for the formation of ZnO particles in propanol, without the addition of a polymer such as PVP [82,83].

The large growth rates obtained here can be explained by the occurrence of depletion forces [84,85], as observed during ZnS particle formation in the presence of PVP, where cluster growth via depletion forces appeared as soon as the particle surface was too crowded by PVP [86].

While large parts of the ZnO cluster growth phase can reasonably be described by this power law approach, neither the nucleation phase nor the growth deceleration can be described. Furthermore, the occurrence of depletion forces is not considered in this model. Thus, to describe the sigmoidal time evolution of *R*^3^(t), the two-step Finke–Watzky (FW) model was used. In this model slow, continuous nucleation $\left(A\overset{{\;k}_{1\;}\;}{\to }B\right)$ is followed by autocatalytic growth $\left(A+B\overset{{\;k}_{2\;}}{\to }2B\right)$ with the nucleation (*k*_1_) and growth (*k*_2_) constants [62,63,64]. Here, the cube of the radius as a function of time is given by [65]

(9) $$t\geq t_{ind}\;\;\;\;\;\;\;{R\left(t\right)}_{FW}^{3}=R_{f}^{3}\cdot \left(1-\frac{k_{1}+k_{2}\cdot {\left[A\right]}_{0}}{k_{2}\cdot {\left[A\right]}_{0}+k_{1}\;e^{{(k}_{1}+k_{2}\cdot {\left[A\right]}_{0})t}}\right)$$

where *R_f_* corresponds to the final cluster size, and [*A*]_0_ to the starting concentration of Zn^2+^. The induction time, here related to the time after which agglomeration of the ZnO particles into clusters is accelerated, is defined by the intersection of the initial and maximal slope and can be determined according to [65]:

(10) $$t_{ind}=\frac{k_{1}+k_{2}\cdot {\left[A\right]}_{0}}{{{(k}_{1}-k_{2}\cdot {\left[A\right]}_{0})}^{2}}\cdot \ln\left(\frac{k_{2}\cdot {\left[A\right]}_{0}}{k_{1}\;}\right)+\frac{2}{k_{1}-k_{2}\cdot {\left[A\right]}_{0}}.$$

Furthermore, the ratio of the growth to nucleation rate constant $S=\frac{k_{2}}{k_{1}\;}$ (M^−1^) is a measure of kinetic control, indicating whether nucleation of new particles or growth of the existing clusters is preferred. Thus, larger *S* values yield narrow size distributions. In addition, larger ratios were shown to be related to larger clusters [87]. Note that the two-step FW model can be extended to include bimolecular aggregation (*B* + *B*→*C*) [88] and autocatalytic agglomeration (*B* + *C*→1.5 *C*) [89,90]. However, this was not performed here to keep the number of free-fitting parameters at a minimum.

As can be seen from the solid lines in Figure 5*,* the FW model provides a quantitative description of the time evolution of the ZnO cluster size for *t* ≥ *t*_ind_. The systematic mismatch of the model at *t* < *t*_ind_ could be due, on one hand, to a nucleation rate that is not constant during the heating process but increases strongly. On the other hand, the spherical form factor used in the analysis of the scattering data represents only an approximation, especially for clusters consisting of a few ZnO particles. The obtained parameters, summarized in Table 2, reflect what was already evident from the USAXS data: the induction time shortens from *t*_ind_ = 27 min at *T* = 58 °C to *t*_ind_ = 20 min for *T* = 63 °C, while the growth rate constant *k*_2_ increases considerably by a factor of 50% at higher temperatures to *k*_2_[A]_0_ = 0.17 s^−1^. The nucleation rate, however, does not change. Consequently, the ratio of the growth-to-nucleation rate constant *S* is higher for *T* = 63 °C, which also confirms the previously mentioned assumption of a faster growth rate for higher temperatures.

The FW model assumes continuous nucleation of ZnO particles. This can be indeed observed in the volume fraction profile of the ZnO particles (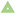) for *T* = 58 °C in Figure 6, where an initial increase is followed by reaching an almost constant value, while the volume fraction of the cluster (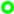) follows a sigmoidal profile. These results suggest that new ZnO particles are formed continuously throughout the reaction, feeding the clusters, which in turn grow simultaneously. Hence, at intermediate times, an equilibrium is established between ZnO particles’ formation and consumption.

Since *R*^3^ is proportional to the volume fraction, Equation (9) can also be used to model *ϕ*_ZnO-cluster_(*t*)*_I_*_(*q*)_ (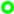) (see solid line in Figure 6), yielding nearly identical values for the FW parameters as compared to the *R*^3^(*t*)-data with *k*_1_(*T* = 58 °C) = 8.5 × 10^−4^ 1/s, *k*_2_[A]_0_ (*T* = 58 °C) = 0.11 1/s with *t*_ind_(*T* = 58 °C) = 29 min.

In a complementary approach, we determined the volume fraction of ZnO clusters *ϕ*_ZnO-cluster_ from the shape- and size-independent scattering invariant *Q* of the time-resolved desmeared USAXS scattering curves for *q* ≤ 0.04 Å^−1^. It should be noted that the invariant was only determined in the low *q*-part, since on the one hand, the scattering in the large *q*-part is dominated by the scattering of PVP, and on the other hand, the USAXS data are noisy in this range. To account only for the scattering of the ZnO clusters, the scattering invariant determined for *t* = 1 min was subtracted from all other data, resulting in a reduced invariant Q*, according to

(11) $$Q^{*}=Q\left(t\right)-Q\left(t=1\;min\right)=\int_{0}^{0.04{Å}^{-1}}q^{2}I\left(q\right)dq=2\pi^{2}{\left(∆\rho \right)}^{2}\phi_{ZnO-cluster}(1-\phi_{ZnO-cluster})$$

where $∆\rho^{2}$ = |*ρ*_ZnO_ − *ρ*_MeOH_ |^2^. In Figure 6, the obtained cluster volume fraction *ϕ*_ZnO-cluster,inv_(*t*) (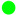) for *T* = 58 °C is plotted as a function of time. Comparing the cluster volume fractions obtained from the model analysis of the USAXS data and the invariant, i.e., *ϕ*_ZnO-cluster,*I*(*q*)_ and *ϕ*_ZnO-cluster,inv_, slightly lower values are found for the latter, which might be a consequence of the finite *q*-range used for the invariant determination as well as the approximation of spherical clusters with compact packing of ZnO particles and a homogeneous scattering length density distribution.

The profiles of the cluster volume fraction *ϕ*_ZnO-cluster,inv_(*t*) were also described using the FW model (in Figure S5 of the Supplementary Materials, the profiles obtained for *T* = 58 °C and *T* = 63 °C are shown for comparison). A comparison of the parameters (Table 2) obtained from the analysis of the *R*^3^(*t*)-data and *ϕ*_ZnO-cluster,inv_(*t*)-data, respectively, reveal the same growth rate constants *k*_2_ and induction times *t*_ind_, which are similar within the measurement uncertainty. The smaller value of the nucleation rate constant for the *ϕ*_ZnO-cluster,inv_(*t*)-data might also be a consequence of the finite *q*-range used for the invariant determination. Consequently, larger *S*-values in the analysis of the *ϕ*_ZnO-cluster,inv_(*t*)-data are obtained.

**Figure 7:**
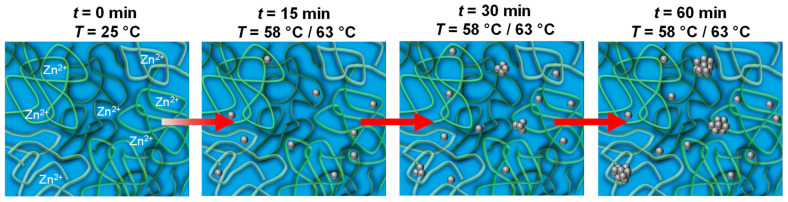
Schematic illustration of the ZnO formation through CBD starting from a methanolic ZnO precursor solution. The organic additive PVP (green strings) forms an entangled network in which the zinc ions are homogeneously distributed. Upon heating, ZnO particles (grey spheres) are continuously formed, which eventually aggregate into clusters.

To conclude the in situ study of ZnO particle formation and growth, we found that shortly after heating the precursor solution, ZnO nanoparticles of the order of *R*_ZnO_ ≈ 1.5 nm are initially formed, followed by the formation of clusters. An increase in the reaction temperature from 58 °C to 63 °C resulted in the acceleration of the reaction and the formation of larger clusters. From the time evolution of the cube of radius, a linear relationship is apparent at intermediate times. Depletion forces caused by the high volume fraction of PVP might explain the relatively high growth rate. A quantitative description of the time evolution of the ZnO nanoparticle cluster radius, the corresponding cube of the radius, as well as the volume fraction, could be achieved by the Finke–Watzky model [62,63,64,65] with slow, continuous nucleation of individual ZnO particles followed by autocatalytic growth of the clusters, as illustrated in Figure 7. From the analysis of the kinetic data, the higher ratio of growth to nucleation rate explains that slightly larger clusters are formed at the higher temperature of 63 °C.

## 4. Conclusions

A crucial step for the synthesis of metal oxide inverse opals is the mineralization of the voids of a close-packed polymer nanoparticle templates via the chemical bath deposition (CBD) process. Intrigued by the vision of tuning the size and shape of ZnO particles, we aimed to gain more insight into the particle formation in an additive containing precursor solution by in situ time-resolved ultra-small- and small-angle X-ray scattering (USAXS/SAXS).

First, we used in-house SAXS and USAXS/SAXS to study the structure of polyvinylpyrrolidone (PVP) applied as structure directing agent for the ZnO formation in the methanolic precursor solution at *T* = 25 °C. We found that at the volume fraction of *ϕ*_PVP_ = 7.1 × 10^−2^ used in the CBD process, the polymer forms a network of entangled chains in which ions of the ZnO precursor salt and the organic base are distributed, causing the polymer chains to adopt a semiflexible behavior.

We then followed the ZnO formation using in situ USAXS/SAXS by heating the precursors solution from *T* = 25 °C to *T* = 58 °C and 63 °C, respectively. A significant increase in the intensity of the forward scattering and the formation of an additional weak shoulder was observed after 13 min, both of which occur more rapidly at *T* = 63 °C. The origin of the two scattering contributions was clarified from post-synthesis TEM images, which clearly showed the presence of two ZnO species: ZnO nanoparticles with a radius of *R*_ZnO,TEM_ = 1.5 ± 0.5 nm and clusters of these with *R*_ZnO-cluster,TEM_ = 8.5 ± 1.4 nm and *R*_ZnO-cluster,TEM_ = 9.7 ± 1.9 nm at *T* = 58 °C and 63 °C, respectively. Through this additional information, we were able to quantitatively analyze the time-resolved USAXS/SAXS data by additively combining the scattering contribution of the PVP network with the scattering contributions of the individual ZnO particles and their clusters. This analysis revealed that the radius of the formed individual ZnO particles (*R*_ZnO,f_ = 1.5 ± 0.3 nm) remains constant over time for both temperatures, while the radius of the particle clusters increases from the induction time onwards to *R*_ZnO-cluster,f_ = 10.5 ± 1.9 nm at *T* = 58 °C and*, R*_ZnO-cluster,f_ = 11.8 ± 2.1 nm at *T* = 63 °C, with *R*^3^(*t*) following a sigmoidal profile.

From the linear relationship of *R*^3^(*t*) at intermediate times, relatively large rate cluster growth constants were determined, which might indicate PVP-induced depletion forces. Finally, using the Finke–Watzky two-step model, we were able to quantitatively describe the ZnO formation with slow, continuous nucleation of individual ZnO particles followed by autocatalytic growth of the clusters. A faster growth rate of the clusters compared to the nucleation of new ZnO particles was observed at higher temperatures, which thus explains the formation of larger clusters at 63 °C.

The determination of the kinetics of ZnO formation, synthesized by CBD using a methanolic solution and PVP as a stabilizing and structuring agent, will provide us with valuable insights into the understanding of ZnO deposition in the voids of densely packed polymer nanoparticle templates, where both surface and confinement effects are expected.

## Figures and Tables

**Table 2:** Values for the nucleation rate *k*_1_ and growth rate constant *k*_2_[A]_0_ determined from the two-step Finke–Watzky model [62,63,64,65] together with the induction time *t*_ind_ and the ratio of the rate constants *S* obtained from the size evolution profile *R^3^*(*t*)_FW_ and the volume fractions from the invariant analysis *ϕ*_ZnO-cluster, inv_(*t*)_FW_. We estimate the relative errors to be about 10%.

	*R^3^*(*t*)_FW_				*ϕ*_ZnO-cluster, inv_(*t*)_FW_			
	*k*_1_*/*s^−1^	*k*_2_[A]_0_/s^−1^	*t*_ind_/min	*S*/M^−1^	*k*_1_*/*s^−1^	*k*_2_[A]_0_/s^−1^	*t*_ind_/min	*S/*M^−1^
58 °C	8.0 × 10^−4^	0.11	27	141	5.5 × 10^−4^	0.11	31	200
63 °C	8.0 × 10^−4^	0.17	20	213	5.0 × 10^−4^	0.17	23	340

## Data Availability

The data presented in this study are available on request from the corresponding author.

## Supplementary Materials

The following supporting information can be downloaded at https://www.mdpi.com/article/10.3390/nano13152180/s1: SAXS fitting parameters of PVP in methanol at *ϕ*_PVP_ = 7.1 × 10^−2^ before and after adding 12.5 mM ZnAc_2_ and 26.3 mM TEAOH obtained with the polymer with excluded volume model (Table S1) or the correlation length model (Table S2) combined with a Gaussian, respectively; Figure S1: Slit-smeared SAXS curves of PVP in methanol at various volume fractions *ϕ*; SAXS fitting parameters from PVP in methanol at *ϕ*_PVP_ = 1.0, 2.0, 4.0, and 7.1 × 10^−2^ obtained with the polymer with excluded volume model (Table S3) and the correlation length model (Table S4) combined with a Gaussian, respectively. Figure S2: Obtained fitting parameters for PVP in methanol at various volume fractions *ϕ*_PVP_; Figure S3: Final fit for the scattering curve of the precursor solution after heating to *T* = 58 °C for *t* = 87 min with the respective scattering contributions. Figure S4: TEM image taken after the ZnO synthesis for *T* = 63 °C with the corresponding volume-weighted size distribution of the ZnO particles and clusters, described by a Gaussian size distribution, respectively. Figure S5: Time evolution of the volume fraction *ϕ*_ZnO-cluster,inv_ of ZnO particle clusters determined from the invariant analysis (*ϕ*_invariant_) of the USAXS curves up to *q* ≤ 0.04 Å^−1^. The profiles were described by the two-step Finke–Watzky model (solid lines) from which the induction time *t*_ind_ (stars) were calculated.

## References

[1] Sanchez C., Belleville P., Popall M., Nicole L. (2011). Applications of advanced hybrid organic–inorganic nanomaterials: From laboratory to market. Chem. Soc. Rev..

[2] Sanchez C., Julián B., Belleville P., Popall M. (2005). Applications of hybrid organic–inorganic nanocomposites. J. Mater. Chem..

[3] Mir S.H., Nagahara L.A., Thundat T., Mokarian-Tabari P., Furukawa H., Khosla A. (2018). Review—Organic-Inorganic Hybrid Functional Materials: An Integrated Platform for Applied Technologies. J. Electrochem. Soc..

[4] Eder D. (2010). Carbon Nanotube−Inorganic Hybrids. Chem. Rev..

[5] Khan F.S.A., Mubarak N.M., Khalid M., Khan M.M., Tan Y.H., Walvekar R., Abdullah E.C., Karri R.R., Rahman M.E. (2021). Comprehensive review on carbon nanotubes embedded in different metal and polymer matrix: Fabrications and applications. Crit. Rev. Solid State Mater. Sci..

[6] Zou H., Wu S., Shen J. (2008). Polymer/Silica Nanocomposites: Preparation, Characterization, Properties, and Applications. Chem. Rev..

[7] Adnan M.M., Dalod A.R.M., Balci M.H., Glaum J., Einarsrud M.-A. (2018). In Situ Synthesis of Hybrid Inorganic–Polymer Nanocomposites. Polymers.

[8] Shenton W., Douglas T., Young M., Stubbs G., Mann S. (1999). Inorganic-Organic Nanotube Composites from Template Mineralization of Tobacco Mosaic Virus. Adv. Mater..

[9] Lee S.-Y., Lim J.-S., Harris M.T. (2012). Synthesis and application of virus-based hybrid nanomaterials. Biotechnol. Bioeng..

[10] Caruso R.A., Antonietti M. (2001). Sol−Gel Nanocoating: An Approach to the Preparation of Structured Materials. Chem. Mater..

[11] Lei Q., Guo J., Kong F., Cao J., Wang L., Zhu W., Brinker C.J. (2021). Bioinspired Cell Silicification: From Extracellular to Intracellular. J. Am. Chem. Soc..

[12] Parikh H., de Guire M.R. (2009). Recent progress in the synthesis of oxide films from liquid solutions. J. Ceram. Soc. Jpn..

[13] Koczkur K.M., Mourdikoudis S., Polavarapu L., Skrabalak S.E. (2015). Polyvinylpyrrolidone (PVP) in nanoparticle synthesis. Dalton Trans..

[14] Carreón-Moncada I., González L., Rodríguez-Galicia J., Rendón-Angeles J. (2016). Chemical deposition of CdS films by an ammonia-free process with amino acids as complexing agents. Thin Solid Film..

[15] Enríquez J.P., Mathew X. (2003). Influence of the thickness on structural, optical and electrical properties of chemical bath deposited CdS thin films. Sol. Energy Mater. Sol. Cells.

[16] Lee J.-H. (2006). Structural and optical properties of CdS thin films on organic substrates for flexible solar cell applications. J. Electroceramics.

[17] Preda N., Enculescu M., Gherendi F., Matei E., Toimil-Molares M., Enculescu I. (2012). Synthesis of CdS nanostructures using template-assisted ammonia-free chemical bath deposition. J. Phys. Chem. Solids.

[18] Shimizu K., Imai H., Hirashima H., Tsukuma K. (1999). Low-temperature synthesis of anatase thin films on glass and organic substrates by direct deposition from aqueous solutions. Thin Solid Film..

[19] Santhiya D., Burghard Z., Greiner C., Jeurgens L.P.H., Subkowski T., Bill J. (2010). Bioinspired Deposition of TiO_2_ Thin Films Induced by Hydrophobins. Langmuir.

[20] Dussan A., Bohórquez A., Quiroz H.P. (2017). Effect of annealing process in TiO_2_ thin films: Structural, morphological, and optical properties. Appl. Surf. Sci..

[21] O’Brien P., Saeed T., Knowles J. (1996). Speciation and the nature of ZnO thin films from chemical bath deposition. J. Mater. Chem..

[22] Tseng Y.-H., Lin H.-Y., Liu M.-H., Chen Y.-F., Mou C.-Y. (2009). Biomimetic Synthesis of Nacrelike Faceted Mesocrystals of ZnO−Gelatin Composite. J. Phys. Chem. C.

[23] Shi Z., Walker A.V. (2015). Chemical Bath Deposition of ZnO on Functionalized Self-Assembled Monolayers: Selective Deposition and Control of Deposit Morphology. Langmuir.

[24] Fortunato E., Barquinha P., Pimentel A., Gonçalves A., Marques A., Pereira L., Martins R. (2005). Recent advances in ZnO transparent thin film transistors. Thin Solid Film..

[25] Gordillo G., Pena J. (2022). Development of system to grow ZnO films by plasma assisted reactive evaporation with improved thickness homogeneity for using in solar cells. J. Mater. Res. Technol..

[26] Zhang S., Li H., Wang X., Liu Y., Dai J., Chen C. (2020). Highly Integrated In Situ Photoenergy Gas Sensor with Deep Ultraviolet LED. ACS Omega.

[27] Ahn M.-W., Park K.-S., Heo J.-H., Park J.-G., Kim D.-W., Choi K.J., Lee J.-H., Hong S.-H. (2008). Gas sensing properties of defect-controlled ZnO-nanowire gas sensor. Appl. Phys. Lett..

[28] Ryu Y.R., Lubguban J.A., Lee T.S., White H.W., Jeong T.S., Youn C.J., Kim B.J. (2007). Excitonic ultraviolet lasing in ZnO-based light emitting devices. Appl. Phys. Lett..

[29] Dong H., Zhou B., Li J., Zhan J., Zhang L. (2017). Ultraviolet lasing behavior in ZnO optical microcavities. J. Mater..

[30] Lipowsky P., Jia S., Hoffmann R.C., Jin-Phillipp N.Y., Bill J., Rühle M. (2006). Thin film formation by oriented attachment of polymer-capped nanocrystalline ZnO. Int. J. Mater. Res..

[31] Lipowsky P., Hoffmann R.C., Welzel U., Bill J., Aldinger F. (2007). Site-Selective Deposition of Nanostructured ZnO Thin Films from Solutions Containing Polyvinylpyrrolidone. Adv. Funct. Mater..

[32] Lipowsky P., Burghard Ž., Jeurgens L.P.H., Bill J., Aldinger F. (2007). Laminates of zinc oxide and poly(amino acid) layers with enhanced mechanical performance. Nanotechnology.

[33] Lipowsky P., Hedin N., Bill J., Hoffmann R.C., Ahniyaz A., Aldinger F., Bergström L. (2008). Controlling the Assembly of Nanocrystalline ZnO Films by a Transient Amorphous Phase in Solution. J. Phys. Chem. C.

[34] Lipowsky P., Hirscher M., Hoffmann R.C., Bill J., Aldinger F. (2007). Zinc oxide microcapsules obtained via a bio-inspired approach. Nanotechnology.

[35] Qawasmi Y., Atanasova P., Jahnke T., Burghard Z., Müller A., Grassberger L., Strey R., Bill J., Sottmann T. (2018). Synthesis of nanoporous organic/inorganic hybrid materials with adjustable pore size. Colloid Polym. Sci..

[36] Atanasova P., Weitz R.T., Gerstel P., Srot V., Kopold P., van Aken P.A., Burghard M., Bill J. (2009). DNA-templated synthesis of ZnO thin layers and nanowires. Nanotechnology.

[37] Atanasova P., Rothenstein D., Schneider J.J., Hoffmann R.C., Dilfer S., Eiben S., Wege C., Jeske H., Bill J. (2011). Virus-Templated Synthesis of ZnO Nanostructures and Formation of Field-Effect Transistors. Adv. Mater..

[38] Atanasova P., Stitz N., Sanctis S., Maurer J.H.M., Hoffmann R.C., Eiben S., Jeske H., Schneider J.J., Bill J. (2015). Genetically Improved Monolayer-Forming Tobacco Mosaic Viruses to Generate Nanostructured Semiconducting Bio/Inorganic Hybrids. Langmuir.

[39] Stitz N., Eiben S., Atanasova P., Domingo N., Leineweber A., Burghard Z., Bill J. (2016). Piezoelectric Templates—New Views on Biomineralization and Biomimetics. Sci. Rep..

[40] Atanasova P., Hoffmann R.C., Stitz N., Sanctis S., Burghard Z., Bill J., Schneider J.J., Eiben S. (2019). Engineered nanostructured virus/ZnO hybrid materials with dedicated functional properties. Bioinspired Biomim. Nanobiomaterials.

[41] Abitaev K., Qawasmi Y., Atanasova P., Dargel C., Bill J., Hellweg T., Sottmann T. (2021). Adjustable polystyrene nanoparticle templates for the production of mesoporous foams and ZnO inverse opals. Colloid Polym. Sci..

[42] Kousik S.R., Sipp D., Abitaev K., Li Y., Sottmann T., Koynov K., Atanasova P. (2021). From Macro to Mesoporous ZnO Inverse Opals: Synthesis, Characterization and Tracer Diffusion Properties. Nanomaterials.

[43] Lin X., Chen M. (2016). Fabrication and Photo-Detecting Performance of 2D ZnO Inverse Opal Films. Appl. Sci..

[44] Kiyomi Y., Shiraiwa N., Nakazawa T., Fukawa A., Oshio K., Takase K., Ito T., Shingubara S., Shimizu T. (2022). Fabrication and UV photoresponse of ordered ZnO nanonets using monolayer colloidal crystal template. Micro Nano Eng..

[45] Li Q., Yang C. (2017). Facile fabrication of Ag_3_PO_4_ supported on ZnO inverse opals for enhancement of solar-driven photocatalysis. Mater. Lett..

[46] Meldrum F.C., Cölfen H. (2008). Controlling Mineral Morphologies and Structures in Biological and Synthetic Systems. Chem. Rev..

[47] Rieger J., Kellermeier M., Nicoleau L. (2014). Die Bildung von Nanopartikeln und Nanostrukturen—CaCO_3_, Zement und Polymere aus Sicht der Industrie. Angew. Chem..

[48] Caetano B.L., Santilli C.V., Pulcinelli S.H., Briois V. (2011). SAXS and UV–Vis combined to Quick-XAFS monitoring of ZnO nanoparticles formation and growth. Phase Transit..

[49] Caetano B.L., Santilli C.V., Meneau F., Briois V., Pulcinelli S.H. (2011). In Situ and Simultaneous UV−vis/SAXS and UV−vis/XAFS Time-Resolved Monitoring of ZnO Quantum Dots Formation and Growth. J. Phys. Chem. C.

[50] Caetano B.L., Briois V., Pulcinelli S.H., Meneau F., Santilli C.V. (2017). Revisiting the ZnO Q-dot Formation Toward an Integrated Growth Model: From Coupled Time Resolved UV–Vis/SAXS/XAS Data to Multivariate Analysis. J. Phys. Chem. C.

[51] Penn R.L., Banfield J.F. (1998). Imperfect Oriented Attachment: Dislocation Generation in Defect-Free Nanocrystals. Science.

[52] Ribeiro C., Lee E.J.H., Longo E., Leite E.R. (2005). A Kinetic Model to Describe Nanocrystal Growth by the Oriented Attachment Mechanism. ChemPhysChem.

[53] Lifshitz I., Slyozov V. (1961). The kinetics of precipitation from supersaturated solid solutions. J. Phys. Chem. Solids.

[54] Wagner C. (1961). Theorie der Alterung von Niederschlägen durch Umlösen (Ostwald-Reifung). Z. Electrochem..

[55] Herbst M., Hofmann E., Förster S. (2019). Nucleation and Growth Kinetics of ZnO Nanoparticles Studied by in Situ Microfluidic SAXS/WAXS/UV–Vis Experiments. Langmuir.

[56] Biswas K., Das B., Rao C.N.R. (2008). Growth Kinetics of ZnO Nanorods: Capping-Dependent Mechanism and Other Interesting Features. J. Phys. Chem. C.

[57] Orthaber D., Bergmann A., Glatter O. (2000). SAXS experiments on absolute scale with Kratky systems using water as a secondary standard. J. Appl. Crystallogr..

[58] Ilavsky J., Jemian P.R., Allen A.J., Zhang F., Levine L.E., Long G.G. (2009). Ultra-small-angle X-ray scattering at the Advanced Photon Source. J. Appl. Crystallogr..

[59] Ilavsky J., Zhang F., Andrews R.N., Kuzmenko I., Jemian P.R., Levine L.E., Allen A.J. (2018). Development of combined microstructure and structure characterization facility for in situ and *operando* studies at the Advanced Photon Source. J. Appl. Crystallogr..

[60] Ilavsky J. (2012). Nika: Software for two-dimensional data reduction. J. Appl. Crystallogr..

[61] Ilavsky J., Jemian P.R. (2009). Irena: Tool suite for modeling and analysis of small-angle scattering. J. Appl. Crystallogr..

[62] Watzky M.A., Finke R.G. (1997). Transition Metal Nanocluster Formation Kinetic and Mechanistic Studies. A New Mechanism When Hydrogen Is the Reductant: Slow, Continuous Nucleation and Fast Autocatalytic Surface Growth. J. Am. Chem. Soc..

[63] Finney E.E., Finke R.G. (2008). Nanocluster nucleation and growth kinetic and mechanistic studies: A review emphasizing transition-metal nanoclusters. J. Colloid Interface Sci..

[64] Finney E.E., Finke R.G. (2009). Is There a Minimal Chemical Mechanism Underlying Classical Avrami-Erofe’ev Treatments of Phase-Transformation Kinetic Data?. Chem. Mater..

[65] Bentea L., Watzky M.A., Finke R.G. (2017). Sigmoidal Nucleation and Growth Curves Across Nature Fit by the Finke–Watzky Model of Slow Continuous Nucleation and Autocatalytic Growth: Explicit Formulas for the Lag and Growth Times Plus Other Key Insights. J. Phys. Chem. C.

[66] Timaeva O., Pashkin I., Mulakov S., Kuzmicheva G., Konarev P., Terekhova R., Sadovskaya N., Czakkel O., Prevost S. (2020). Synthesis and physico-chemical properties of poly(N-vinyl pyrrolidone)-based hydrogels with titania nanoparticles. J. Mater. Sci..

[67] Benoit H. (1957). The diffusion of light by polymers dissolved in a good solvent. Comptes Rendus.

[68] Hammouda B. (1993). SANS from homogeneous polymer mixtures: A unified overview. Polymer Characteristics.

[69] Hammouda B., Ho D.L., Kline S. (2004). Insight into Clustering in Poly(ethylene oxide) Solutions. Macromolecules.

[70] Pedersen J.S., Schurtenberger P. (1996). Scattering Functions of Semiflexible Polymers with and without Excluded Volume Effects. Macromolecules.

[71] Chen W.-R., Butler P.D., Magid L.J. (2006). Incorporating Intermicellar Interactions in the Fitting of SANS Data from Cationic Wormlike Micelles. Langmuir.

[72] Porod G. (1951). Die Röntgenkleinwinkelstreuung von dichtgepackten kolloiden Systemen. Kolloid Zeit..

[73] Sapir L., Stanley C.B., Harries D. (2016). Properties of Polyvinylpyrrolidone in a Deep Eutectic Solvent. J. Phys. Chem. A.

[74] Hamada F., Kinugasa S., Hayashi H., Nakajima A. (1985). Small-angle x-ray scattering from semidilute polymer solutions. I. Polystyrene in toluene. Macromolecules.

[75] Distaso M., Taylor R.N.K., Taccardi N., Wasserscheid P., Peukert W. (2011). Influence of the Counterion on the Synthesis of ZnO Mesocrystals under Solvothermal Conditions. Chem.-A Eur. J..

[76] Knappe P., Bienert R., Weidner S., Thünemann A.F. (2010). Characterization of poly(N-vinyl-2-pyrrolidone)s with broad size distributions. Polymer.

[77] Pavlov G.M., Panarin E.F., Korneeva E.V., Kurochkin C.V., Baikov V.E., Ushakova V.N. (1990). Hydrodynamic properties of poly(1-vinyl-2-pyrrolidone) molecules in dilute solution. Makromol. Chem..

[78] Benoit H., Doty P. (1953). Light Scattering from Non-Gaussian Chains. J. Phys. Chem..

[79] Narayanan T., Sztucki M., Van Vaerenbergh P., Léonardon J., Gorini J., Claustre L., Sever F., Morse J., Boesecke P. (2018). A multipurpose instrument for time-resolved ultra-small-angle and coherent X-ray scattering. J. Appl. Crystallogr..

[80] Guinier A., Fournet G. (1955). Small-Angle Scattering of X-rays.

[81] Jouault N., Crawford M.K., Chi C., Smalley R.J., Wood B., Jestin J., Melnichenko Y.B., He L., Guise W.E., Kumar S.K. (2016). Polymer Chain Behavior in Polymer Nanocomposites with Attractive Interactions. ACS Macro Lett..

[82] Wong E.M., Bonevich J.E., Searson P.C. (1998). Growth Kinetics of Nanocrystalline ZnO Particles from Colloidal Suspensions. J. Phys. Chem. B.

[83] Hu Z., Santos J.F.H., Oskam G., Searson P.C. (2005). Influence of the reactant concentrations on the synthesis of ZnO nanoparticles. J. Colloid Interface Sci..

[84] Asakura S., Oosawa F. (1954). On Interaction between Two Bodies Immersed in a Solution of Macromolecules. J. Chem. Phys..

[85] Asakura S., Oosawa F. (1958). Interaction between particles suspended in solutions of macromolecules. J. Polym. Sci..

[86] Ghosh G., Naskar M.K., Patra A., Chatterjee M. (2006). Synthesis and characterization of PVP-encapsulated ZnS nanoparticles. Opt. Mater..

[87] Watzky M.A., Finke R.G. (1997). Nanocluster Size-Control and “Magic Number” Investigations. Experimental Tests of the “Living-Metal Polymer” Concept and of Mechanism-Based Size-Control Predictions Leading to the Syntheses of Iridium(0) Nanoclusters Centering about Four Sequential Magic Numbers. Chem. Mater..

[88] Hornstein B.J., Finke R.G. (2004). Transition-Metal Nanocluster Kinetic and Mechanistic Studies Emphasizing Nanocluster Agglomeration: Demonstration of a Kinetic Method That Allows Monitoring of All Three Phases of Nanocluster Formation and Aging. Chem. Mater..

[89] Besson C., Finney E.E., Finke R.G. (2005). A Mechanism for Transition-Metal Nanoparticle Self-Assembly. J. Am. Chem. Soc..

[90] Besson C., Finney E.E., Finke R.G. (2005). Nanocluster Nucleation, Growth, and Then Agglomeration Kinetic and Mechanistic Studies: A More General, Four-Step Mechanism Involving Double Autocatalysis. Chem. Mater..

