# Supplementary material for: In Situ Ultra-Small- and Small-Angle X-ray Scattering Study of ZnO Nanoparticle Formation and Growth through Chemical Bath Deposition in the Presence of Polyvinylpyrrolidone
Source: Nanomaterials (Basel). 2023 Jul 26;13(15):2180. doi: 10.3390/nano13152180 (PMC10421471; doi:10.3390/nano13152180)
Supplement: Supplementary file 1 [file nanomaterials-13-02180-s001.zip › nanomaterials-2521267-supplementary.pdf]

Supplementary Material

In Situ Ultra-Small- and Small-Angle X-Ray Scattering Study of ZnO Nanoparticle Formation and Growth through Chemical Bath Deposition in the Presence of Polyvinylpyrrolidone

Karina Abitaev [1], Petia Atanasova [2], Joachim Bill [2], Natalie Preisig [1], Ivan Kuzmenko [3], Jan Ilavsky [3], Yun Liu [4] and Thomas Sottmann [1,*]

[1] Institute of Physical Chemistry, University of Stuttgart, 70569 Stuttgart, Germany; karina.abitaev@ipc.uni-stuttgart.de (K.A.); natalie.preisig@ipc.uni-stuttgart.de (N.P.)
[2] Institute for Materials Science, University of Stuttgart, 70569 Stuttgart, Germany; atanasova@imw.uni-stuttgart.de (P.A.); bill@imw.uni-stuttgart.de (J.B.)
[3] X-ray Science Division, Advanced Photon Source, Argonne National Laboratory, Argonne, IL 60439, USA; kuzmenko@anl.gov (I.K.); ilavsky@anl.gov (J.I.)
[4] National Institute of Standards and Technology Center for Neutron Research, Gaithersburg, MD 20899, USA; yun.liu@nist.gov (Y.L)
* Correspondence: thomas.sottmann@ipc.uni-stuttgart.de (T.S)

Table of Contents

1. **Scattering curve analysis for PVP in methanol before and after adding $ZnAc_2$ and TEAOH** 2
2. **USAXS/SAXS curve modelling of the precursor solution after heating** 4
3. **TEM images analysis for T = 63 °C** 5
4. **Invariant based time evolution of the ZnO cluster volume fraction** 5
5. **References** 6

1. Scattering curve analysis for PVP in methanol before and after adding $ZnAc_2$ and TEAOH

Scattering curves from methanolic precursor solutions at constant polyvinylpyrrolidone (PVP) volume fraction $\phi_{PVP} = 7.1 \times 10^{-2}$ before and after adding tetraethylammonium hydroxide (TEAOH) or zinc acetate ($ZnAc_2$) were analyzed according to the polymer with excluded volume model [1, 2] and the correlation length model [3] combined with a Gaussian, respectively, see equation 3 and 4 in the main text. The corresponding fitting parameters are summarized in Table S1 and S2

Table S1: SAXS fitting parameters of PVP in methanol at $\phi_{PVP} = 7.1 \times 10^{-2}$ before and after adding 12.5 mM $ZnAc_2$ and 26.3 mM TEAOH obtained with the polymer with excluded volume model [1, 2] combined with a Gaussian yielded the forward scattering I_0, the radius of gyration R_g(PVP), the Porod exponent m, as well as the Gaussian scaling factor G, the peak position q_G and width σ_G.

Sample	I_0 / cm^{-1}	R_g(PVP) / nm	m	G	q_G/ Å^{-1}	σ_G/ Å^{-1}
PVP	0.53 ± 0.01	1.5 ± 0.1	1.52 ± 0.01	0.018 ± 0.005	1.54 ± 0.01	0.22 ± 0.02
PVP - TEAOH	0.52 ± 0.01	1.8 ± 0.1	1.45 ± 0.01	0.029 ± 0.005	1.54 ± 0.01	0.22 ± 0.02
PVP - $ZnAc_2$	0.50 ± 0.01	1.7 ± 0.1	1.46 ± 0.01	0.020 ± 0.005	1.52 ± 0.01	0.24 ± 0.02

Table S2: SAXS fitting parameters obtained from the analysis of SAXS data from PVP in methanol at $\phi_{PVP} = 7.1 \times 10^{-2}$ before and after adding 12.5 mM $ZnAc_2$ and 26.3 mM TEAOH with the correlation length model [3] combined with a Gaussian. In this context, the Lorentzian scaling factor C, the correlation length ξ_{PVP} and the Lorentzian exponent b were obtained, while the parameters for the Gaussian remained the same as in Table S1.

Sample	A	m'	C	b	ξ_{PVP} / nm
PVP	-	-	0.59 ± 0.01	1.7 ± 0.1	1.1 ± 0.1
PVP - TEAOH	-	-	0.61 ± 0.01	1.5 ± 0.1	1.3 ± 0.1
PVP - $ZnAc_2$	-	-	0.59 ± 0.01	1.5 ± 0.1	1.3 ± 0.1
PVP - TEAOH - $ZnAc_2$	0.007 ± 0.001	1.55 ± 0.01	0.60 ± 0.01	1.5 ± 0.1	2.9 ± 0.2

Furthermore, slit smeared SAXS curves of PVP in methanol at ϕ_{PVP} = 1.0, 2.0, 4.0, and 7.1 × 10^{-2} were recorded at 25 °C and analyzed analogous to the previous scattering curves. The corresponding fitting parameters are summarized in Table S2-4. Note that the solid lines in Figure S1 were obtained by applying the former analysis.

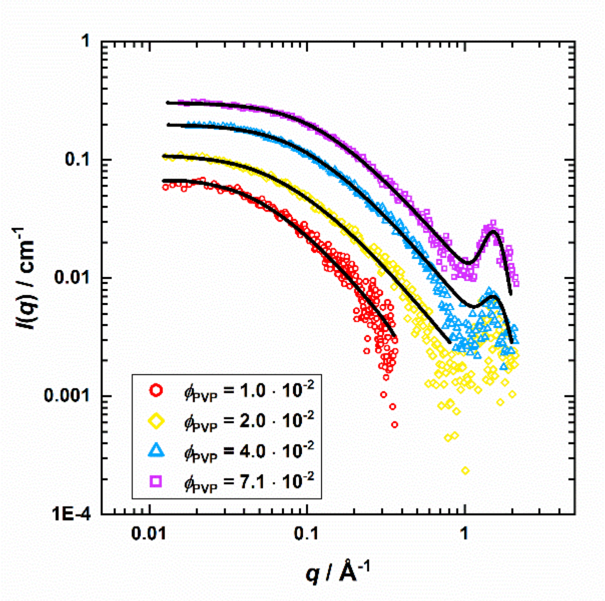

Figure S1: Slit-smeared SAXS curves of PVP in methanol at various volume fractions ϕ_{PVP}. The scattering curves were described using the form factor for polymers with excluded volume effects [1, 2] combined with a Gaussian.

Table S3: SAXS fitting parameters from PVP in methanol at ϕ_{PVP} = 1.0, 2.0, 4.0, and 7.1 × 10^{-2} obtained with the polymer with excluded volume model [1, 2] combined with a Gaussian, yielded the forward scattering I_0, the radius of gyration R_g(PVP), the Porod exponent m, as well as the Gaussian scaling factor G, the peak position q_G and width σ_G.

ϕPVP 10^{-2}	I_0/cm^{-1}	Rg(PVP)/nm	m	G	qG/Å^{-1}	σG/Å^{-1}
1.0	0.25 ± 0.01	3.5 ± 0.1	1.91 ± 0.07	-	-	-
2.0	0.31 ± 0.01	2.9 ± 0.1	1.63 ± 0.01	-	-	-
4.0	0.45 ± 0.01	2.2 ± 0.1	1.60 ± 0.01	0.004 ± 0.002	1.51 ± 0.01	0.22 ± 0.01
7.1	0.53 ± 0.01	1.5 ± 0.1	1.52 ± 0.01	0.020 ± 0.005	1.54 ± 0.01	0.22 ± 0.01

Table S4: Fitting parameters from the analysis of SAXS curves from PVP in methanol at ϕ_{PVP} = 1.0, 2.0, 4.0, and 7.1 × 10^{-2} with the correlation length model [3] combined with a Gaussian. In this context, the scaling factor *C*, the correlation length ξ_{PVP} and the Lorentzian exponent *b* were obtained, while the parameters for the Gaussian are the same as in Table S3.

ϕPVP·10^{-2}	*C*	ξPVP/nm	*b*
1.0	0.22 ± 0.01	2.0 ± 0.1	2.0 ± 0.1
2.0	0.35 ± 0.01	2.0 ± 0.1	1.8 ± 0.1
4.0	0.54 ± 0.01	1.7 ± 0.1	1.7 ± 0.1
7.1	0.59 ± 0.01	1.1 ± 0.1	1.7 ± 0.1

Figure S2 shows the dependency of the polymer characteristics R_g(PVP), the Porod exponent *m*, and the correlation length ξ_{PVP} as a function of the PVP volume fraction.

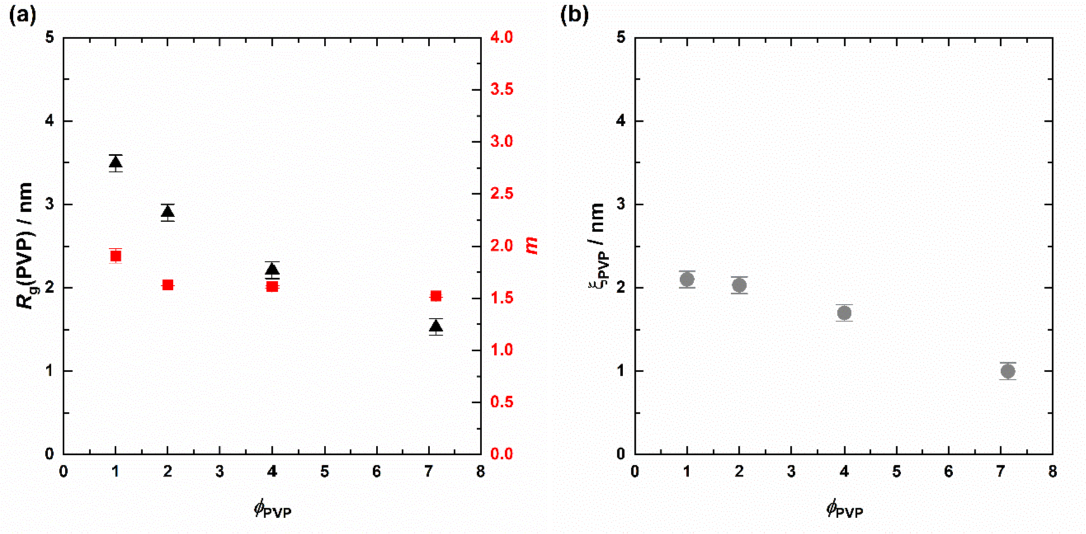

Figure S2: Obtained fitting parameters for PVP in methanol at various volume fractions ϕ_{PVP} using the polymer with excluded volume model [1, 2] with the Guinier radius $\boldsymbol{R_g}$, the Porod exponent *m* (**a**), and the correlation length ξ determined from the correlation length analysis [3] (**b**).

2. USAXS/SAXS curve modelling of the precursor solution after heating

USAXS/SAXS curves obtained after heating the precursor solution were modeled by considering the scattering contribution of (1) the PVP, described by the flexible cylinder model [4, 5], (2) individual ZnO particles and (3) the clusters of these, described by a polydisperse sphere form factor [6], respectively, as well as (4) the C-C correlation peak, described by a Gaussian. The respective contributions as well as the final fit are shown exemplary for T = 58 °C in Figure S3.

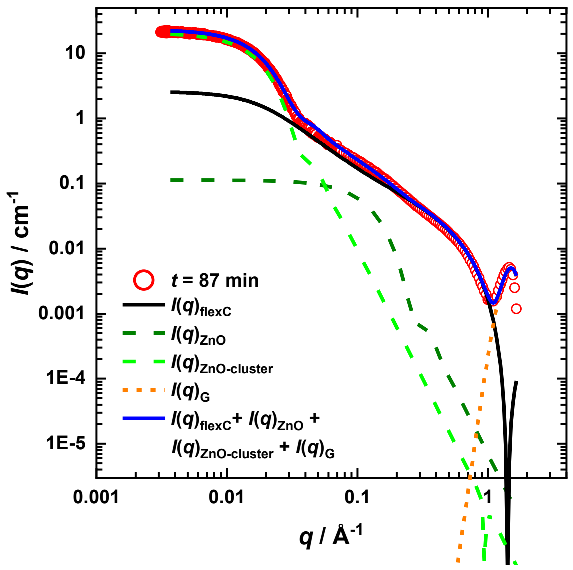

Figure S3: Final scattering curve of the precursor solution after heating to $T = 58$ °C for $t = 87$ min, described by the flexible cylinder model [4, 5] used for $t = 0$ min which was additively combined with two polydisperse sphericalform factors [6] for the scattering contributions of the ZnO particles and their clusters as well as with a Gaussian.

3. TEM images analysis for T = 63 °C

TEM images were taken from a separately prepared solution after an analogous heating procedure starting from 25 °C to 63 °C. An exemplary TEM image as well as the corresponding size distribution of the ZnO particle and cluster size is shown in Figure S4. Therein, ZnO particles with a radius of $R_{ZnO,TEM}$ = 1.7 ± 0.5 nm are clearly visible as well as larger clusters with an average radius of $R_{ZnO\text{-}cluster,TEM}$ = 9.7 ± 1.9 nm.

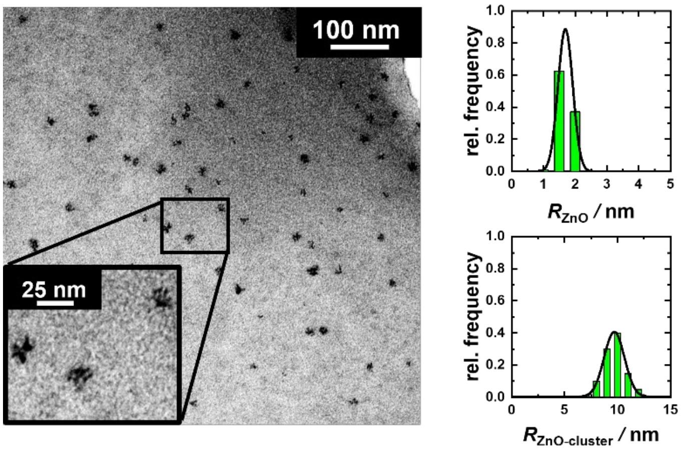

Figure S4: TEM image taken after the ZnO synthesis for T = 63 °C with the corresponding volume-weighted size distribution of the ZnO particles and clusters, described by a Gaussian size distribution, respectively.

4. Invariant based time evolution of the ZnO cluster volume fraction

The scattering invariant of the time-resolved desmeared USAXS scattering curves in the range of $q \le$ 0.04 Å^{-1} was used to determine the volume fraction $f_{ZnO\text{-}cluster}$ of the ZnO clusters as a function of time. These curves were then analyzed by the mechanism-based Finke-Watzky model [7–9]. For more details, see the main text.

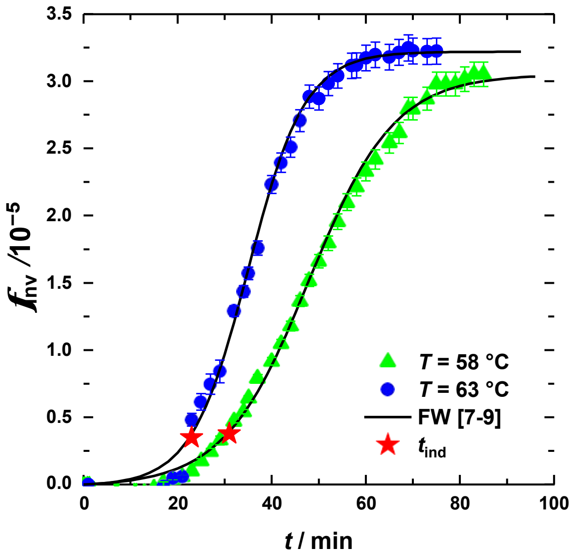

Figure S5: Time evolution of the volume fraction $f_{ZnO\text{-}cluster,inv}$ of ZnO particle clusters determined from the invariant analysis of the USAXS curves up to $q \le$ 0.04 Å^{-1}. The profiles were described by the two-step Finke-Watzky model [7–9] (solid lines) from which the induction time t_{ind} (stars) were calculated.

References

1. Benoit, H. The diffusion of light by polymers dissolved in a good solvent. *Comptes Rendus* **1957**, *245*, 2244–2247.
2. Hammouda, B. SANS from homogeneous polymer mixtures: A unified overview. In *Polymer Characteristics*; Springer: Berlin/Heidelberg, Germany, 1993; pp 87–133.
3. Hammouda, B.; Ho, D.L.; Kline, S. Insight into Clustering in Poly(ethylene oxide) Solutions. *Macromolecules* **2004**, *37*, 6932–6937. https://doi.org/10.1021/ma049623d.
4. Pedersen, J.S.; Schurtenberger, P. Scattering Functions of Semiflexible Polymers with and without Excluded Volume Effects. *Macromolecules* **1996**, *29*, 7602–7612. https://doi.org/10.1021/ma9607630.
5. Chen, W.-R.; Butler, P.D.; Magid, L.J. Incorporating Intermicellar Interactions in the Fitting of SANS Data from Cationic Wormlike Micelles. *Langmuir* **2006**, *22*, 6539–6548. https://doi.org/10.1021/la0530440.
6. Guinier, A.; Fournet, G. *Small-Angle Scattering of X-Rays*; John Wiley & Sons: New York, NY, USA, 1955.
7. Watzky, M.A.; Finke, R.G. Transition Metal Nanocluster Formation Kinetic and Mechanistic Studies. A New Mechanism When Hydrogen Is the Reductant: Slow, Continuous Nucleation and Fast Autocatalytic Surface Growth. *J. Am. Chem. Soc.* **1997**, *119*, 10382–10400. https://doi.org/10.1021/ja9705102.
8. Finney, E.E.; Finke, R.G. Nanocluster nucleation and growth kinetic and mechanistic studies: A review emphasizing transition-metal nanoclusters. *J. Colloid Interface Sci.* **2008**, *317*, 351–374. https://doi.org/10.1016/j.jcis.2007.05.092.
9. Bentea, L.; Watzky, M.A.; Finke, R.G. Sigmoidal Nucleation and Growth Curves Across Nature Fit by the Finke–Watzky Model of Slow Continuous Nucleation and Autocatalytic Growth: Explicit Formulas for the Lag and Growth Times Plus Other Key Insights. *J. Phys. Chem. C* **2017**, *121*, 5302–5312. https://doi.org/10.1021/acs.jpcc.6b12021.
